# The impact of nationwide folic acid fortification on genetic variants associated with conotruncal heart defects

**DOI:** 10.1007/s00439-026-02888-5

**Published:** 2026-10-22

**Authors:** Sarah U. Morton, Enrique Mondragon-Estrada, Rui Qian, Omobola O. Oluwafemi, Kit Sing Au, Hope Northrup, Wendy K. Chung, A. J. Agopian, Tina O. Findley

**Affiliations:** 1 Department of Pediatrics, Boston Children’s Hospital, Harvard Medical School, Boston, MA United States; 2 Division of Newborn Medicine, Department of Pediatrics, Boston Children’s Hospital, Boston, MA United States; 3 Department of Epidemiology, UTHealth Houston School of Public Health, Houston, TX United States; 4 Division of Medical Genetics, Department of Pediatrics, McGovern Medical School at UTHealth Houston, Houston, TX United States; 5 Division of Neonatal-Perinatal Medicine, Department of Pediatrics, McGovern Medical School at UTHealth Houston, Houston, TX United States

## Abstract

Folate deficiency is associated with increased risk of conotruncal heart defects (CTHD), but its interaction with genetic factors remains unclear. Our objective was to investigate genome-wide associations between common genetic variants and birth before versus after universal folic acid fortification among children with CTHD and other heart defects. Sequencing data were available through the Pediatric Cardiac Genomics Consortium, aligned to the human reference genome (GRCh38/hg38), and jointly processed to ensure uniform variant detection and minimize batch effects. Analyses were restricted to individuals with European-inferred genetic ancestry, limiting generalizability to other ancestral groups. Genome-wide association study (GWAS) models compared allele frequencies across fortification eras among all participants (*n* = 1285), the subset with CTHD (*n* = 534), and the remaining individuals with other heart defects (*n* = 751). Functional enrichment was assessed using the Database for Annotation, Visualization and Integrated Discovery (DAVID). Among the full analytic group, eight loci had at least two suggestive variants before compared to after fortification. Among the subset with CTHD, two variants located in *DHRS3* (rs7551703, odds ratio [OR] 2.10, and rs6541043, OR 2.21) and four variants located in *PPARGC1B* showed suggestive association after fortification (OR 4.47–4.51). Enriched biological pathway terms were consistent with cardiac developmental processes. In summary, this study examined the association between folic acid fortification and genetic risk for CTHD and other heart defects and identified associations with fortification era that suggest some shift in risk following fortification. Identified pathways suggest gene-nutrient interactions may modulate cardiac developmental pathways, underscoring the relationship between maternal folate status and cardiovascular development.

## Introduction

Conotruncal heart defects (CTHD) are the most common group of complex congenital heart defects, arising from disrupted development of the cardiac outflow tract (Van Der Linde et al. 2011; Leirgul et al. 2014). Formation of the cardiac outflow tract relies on neural crest migration and outflow tract septation, and CTHD has shown the strongest association with maternal folate status among congenital heart defect subtypes (Feng et al. 2015). Despite this, the etiology of most congenital heart defects remains unclear. While a genetic condition is identified in approximately one-third of people with congenital heart defects, most do not have a clear genetic association and are likely attributable to currently unknown complex etiologies (e.g., polygenic risk, gene-environment interactions) (Hobbs et al. 2006, 2011; Mamasoula et al. 2013; Wang et al. 2013). Maternal dietary deficiencies during the early stages of pregnancy have been thoroughly investigated, including population studies and randomized control trials, suggesting that periconceptional folic acid fortification is associated with decreased risk for the two most common birth defects, neural tube defects and CTHD (Czeizel 1993; Botto et al. 1996; Czeizel et al. 2015; Qu et al. 2020). Based on these findings, the daily intake of prenatal vitamins containing folic acid is recommended for women of reproductive age, starting one to three months before conception. However, in many pregnancies, prenatal vitamin supplementation to prevent birth defects is often not started during this optimal window. Therefore, in 1998, the United States Food and Drug Administration mandated that grains and cereals be fortified with folic acid to prevent folate deficiency during the critical period of embryogenesis. While these measures significantly reduced the incidence of neural tube defects, a more modest impact occurred in congenital heart defects (Bedard et al. 2013; Czeizel et al. 2013), raising the likelihood of a more complex gene-environment relationship between folic acid and CTHD.

Folate plays a crucial role in one-carbon metabolism, nucleotide synthesis, and epigenetic regulation during embryonic development (Rosenquist 2013). Its deficiency impairs the development of cardiac neural crest cells, resulting in structural defects of the cardiac outflow tract, valves, and septum, manifesting as CTHD (Tang et al. 2004; Taparia et al. 2007). However, genetic epidemiology studies investigating the relationship between variants in folate genes in CTHD have been inconclusive (Shaw et al. 2003; Goldmuntz et al. 2008; Zhu et al. 2012). A challenging aspect of these studies has been accounting for maternal folate deficiency and the relatively small study population. The fortification of grains and cereals with folic acid represents a population-level nutritional intervention that altered the prenatal environment, raising the possibility that it modified the penetrance or expressivity of some genetic risk variants. To our knowledge, no study has directly compared genetic variants among children with CTHD born before and after 1998 in North America, during which the blood levels of folate in the general population, including pregnant women, significantly increased due to folic acid fortification (Dietrich et al. 2005).

Genome-wide association studies (GWAS) have identified multiple loci associated with congenital heart defects, yet many effect sizes are small and often inconsistent, with variability in the inclusion of subtypes contributing to study heterogeneity (Hu et al. 2013; Agopian et al. 2014, 2017; Mitchell et al. 2015; Hanchard et al. 2016; Lahm et al. 2021; Theis et al. 2023). Another explanation for this variability is unmodeled gene-environment interaction. Pre- and post-fortification birth cohorts present an opportunity to examine whether the distributions of genetic variation present among individuals with congenital heart defects differed across fortification eras. Our study objective was to address these previous limitations by utilizing a dataset of genomic sequencing data to explore the association of common genetic variants with pre- and post-fortification eras among children born with CTHD in the United States. The rationale for the study was to identify variants present in abundance before or after folic acid fortification, potentially representing risk alleles for CTHD that are sensitive to folate.

## Methods

### Study subjects

Genomic sequencing data were accessed through the NCBI Database of Genotypes and Phenotypes (dbGaP) from the Pediatric Cardiac Genomics Consortium (PCGC) Study (phs001194.v2.p2) dataset. Details in the study design, patient population, and enrollment sites have been previously described (Pediatric Cardiac Genomics Consortium et al. 2013). Types of CTHD lesions in the case population were categorized based on expert opinion and the National Birth Defects Prevention Study (Botto et al. 2007). Our full analytic group included two subsets: individuals with CTHD and individuals with other congenital heart defects that did not have CTHD. CTHD was categorized using the PCGC-derived variable, which includes tetralogy of Fallot with or without pulmonary atresia, truncus arteriosus, D-transposition of the great arteries with or without ventricular septal defect, double outlet right ventricle, and interrupted aortic arch type B. Other cardiac defects (non-conotruncal) included septal defects (atrial septal defect and ventricular septal defect not classified as conotruncal), left-sided obstruction lesions, atrioventricular canal, total anomalous pulmonary veins, and single ventricle defects. Potential etiologic heterogeneity was suspected between these subsets, though both groups were recruited through the same multicenter study network, and individuals with other congenital heart defects share many characteristics with individuals with CTHDs. Importantly, folic acid fortification has been shown to exert differential effects across congenital heart defect subtypes, with the strongest and most consistent associations observed for CTHD.

### Sequencing and quality control

All participants were sequenced through the PCGC using a centralized platform and harmonized quality control. Genome sequencing data were processed jointly to ensure uniform variant detection and minimize batch effects. Raw sequencing reads were aligned to the human reference genome (GRCh38/hg38). Joint variant calling was conducted across all samples, followed by joint genotyping. This approach allowed harmonized detection of single-nucleotide variants and small insertions/deletions (indels) across the combined cohort. Variants underwent standard quality control procedures, including variants that had genotype quality (GQ) > 20 and allele read depth (AD) > 10. Variants located within problematic genomic regions were removed using bedtools (Richter et al. 2020). Additional filters were applied to retain only bi-allelic SNPs with a minor allele frequency > 0.01, Hardy-Weinberg equilibrium p-value > 1 × 10^− 6^. To minimize population stratification and enhance genetic homogeneity, only individuals of predominantly European-inferred ancestry were included in the analysis, reducing the impact of ancestral differences between groups. Ancestry was inferred using EthSEQ (National Academies of Sciences, 2023). LD pruning was performed (50 kb window, step size = 5 variants, r^2^ > 0.2 threshold), and the pruned SNP set was used for principal component analysis.

### Analysis

The birth years of individuals in each cohort were used to categorize them into two time periods: before and after folic acid fortification, which was the primary exposure of interest. To account for a period of transition in the nationwide implementation of FDA policy, patients born in 1997 or earlier were grouped in the pre-fortification period, and patients born in 2000 or later were grouped in the post-fortification period. Patients with birth years of 1998 or 1999 were excluded as consumption of folic acid-fortified food during pregnancy was deemed uncertain. Thus, to investigate whether allele frequencies differed by fortification era among children with CTHD, we first compared allele frequencies between all participants born before and after folic acid fortification among the full analytic group, including CTHD status as a covariate (GWAS Model 1). We then stratified this analysis by CTHD status, first restricting the analysis to individuals with CTHD (GWAS Model 2) and then restricting it to those with other congenital heart defects (GWAS Model 3).

SNP-level genome-wide association analyses were performed using logistic-Firth hybrid regression under an additive genetic model with PLINK v2.0.0-a.6.22LM. Linkage disequilibrium score regression (LDSC) was used to estimate genomic inflation and confirm adequate control for population stratification, with LDSC intercepts guiding selection of the number of principal components to include. The top ten principal components were included as covariates in the GWAS model, as they provided optimal control for potential population stratification based on inspection of the LDSC intercepts. Suggestive statistical significance was defined with a threshold of *p* < 1 × 10^− 5^. All models were examined for variants in a predefined gene set, including *MTHFR*,* MTRR*,* MTR*, and related pathway genes (Lupo et al. 2010).

### Functional enrichment analysis

To investigate biological pathways that may be involved in the etiology of CHDs, we conducted functional over-representation analysis using the Database for Annotation, Visualization and Integrated Discovery (DAVID), version 6.8 (Huang et al. 2009; Sherman et al. 2022). DAVID integrates multiple biological annotation databases, including Gene Ontology (GO), the Kyoto Encyclopedia of Genes and Genomes (KEGG), and Reactome, to evaluate whether predefined functional categories are overrepresented within a user-specified gene list. Analysis were conducted using a rank-based gene selection approach. For each of the three analytic models, genes were ranked according to their association Z-statistic, and the top 100 genes from each model were selected for downstream functional annotation. Using a fixed number of highly ranked genes ensured consistency across analyses while capturing genes showing the strongest evidence of association. Gene identifiers were uploaded to DAVID using Entrez Gene IDs, and enrichment analyses were performed using both the Functional Annotation Chart and Functional Annotation Clustering tools with default parameters (Huang et al. 2009). Enrichment was evaluated across GO biological process, molecular function, and cellular component categories, as well as KEGG and Reactome pathway databases.

To minimize bias associated with gene set composition, enrichment analyses were conducted using a custom background gene list consisting of all genes included in the gene-level association analyses for each model. Statistical significance for enrichment was assessed using a modified Fisher’s exact test with Benjamini-Hochberg false discovery rate (FDR) adjustment as implemented in DAVID (Huang et al. 2009; Sherman et al. 2022). No annotation term retained significance after FDR adjustment in any model. Enrichment scores for annotation clusters were calculated as the -log10 of the geometric mean of the p-values for the terms within each cluster (Huang et al. 2009; Sherman et al. 2022). Visualized enrichment results were prioritized using unadjusted p-values < 0.05 together with fold enrichment (FE) ≥ 2, consistent with previously described exploratory enrichment strategies (Huang et al. 2009; Raggi et al. 2020; Li et al. 2021). Functional annotation clusters with enrichment scores greater than 1.3 (approximately corresponding to a mean p-value < 0.05) were considered to represent meaningful enrichment signals (Huang et al. 2009; Hazelett et al. 2014). For visualization, enriched annotation terms meeting the criteria of nominal *p* < 0.05 and FE ≥ 2 were manually reviewed to remove redundant entries. Terms representing overlapping biological concepts, including parent-child GO relationships or categories supported by highly overlapping gene sets, were consolidated to improve clarity. Enrichment results were summarized using dot plots, with annotation terms shown on the y-axis, -log10 transformed nominal p-values on the x-axis, and dot size reflecting the number of genes contributing to each enriched term.

## Results

**Fig. 1:**
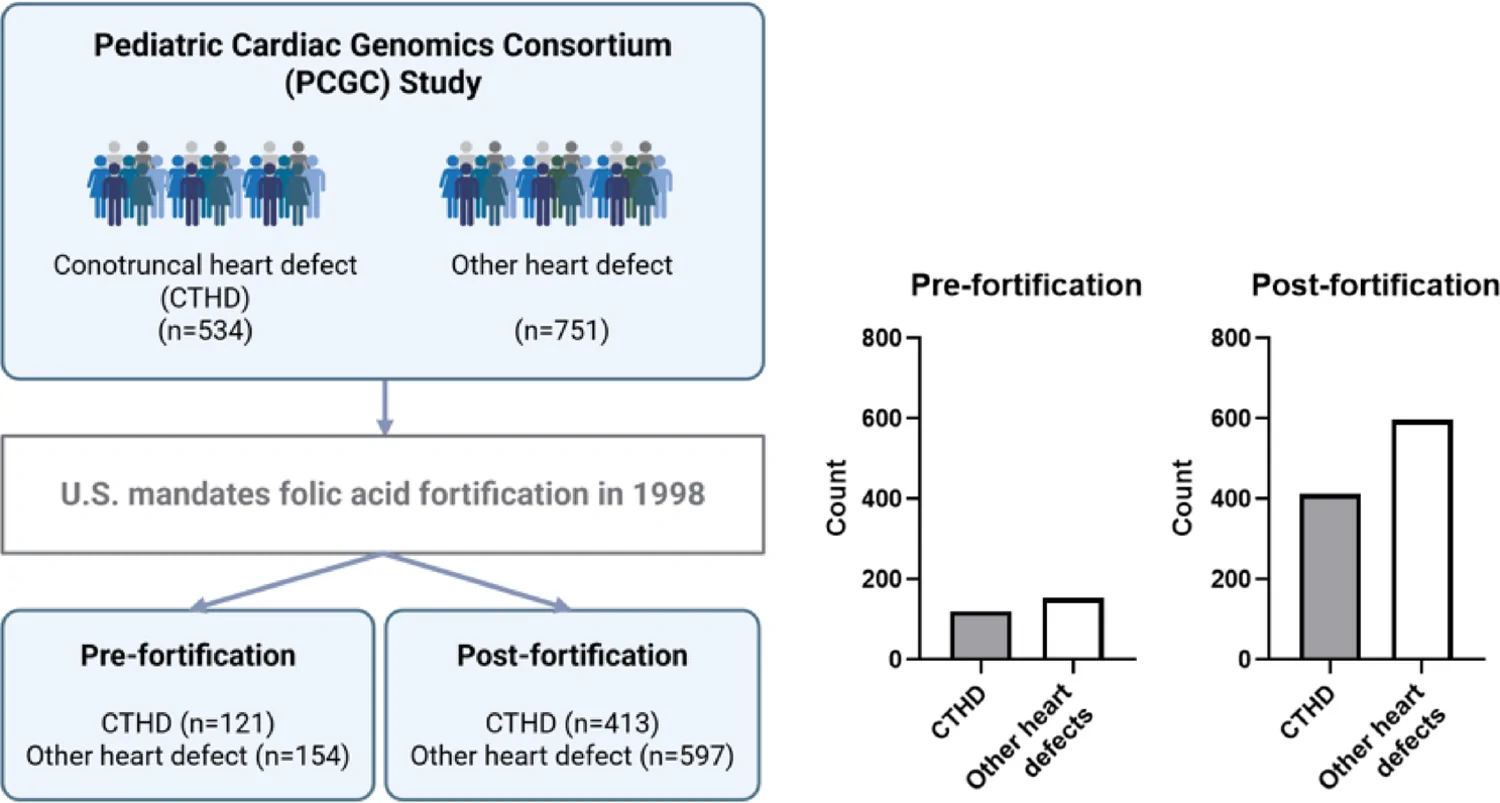
Consort diagram of study participants and genotype data available separated by folate era by European-inferred ancestry

Across the entire PCGC cohort, tetralogy of Fallot was the most common subtype of CTHD (*n* = 1,708; 54.7%), followed by D-transposition of the great arteries (*n* = 845; 27.1%), double outlet right ventricle (*n* = 244; 7.8%), combined double outlet right ventricle with D-transposition of the great arteries (*n* = 70; 2.2%), truncus arteriosus (*n* = 203; 6.5%), and interrupted aortic arch, type B (*n* = 51; 1.6%). Following quality control measures, genotype data were available for 778 individuals with CTHD and 1,111 participants with other congenital heart defects. After restricting to participants of European-inferred ancestry, 534 participants with CTHD (pre-fortification, *n* = 121; post-fortification, *n* = 413) and 751 participants with other heart defects remained (pre-fortification, *n* = 154; post-fortification, *n* = 597) (Fig. 1). The distribution of subtypes are detailed in Supplemental Tables 1 and 2.

**Fig. 2:**
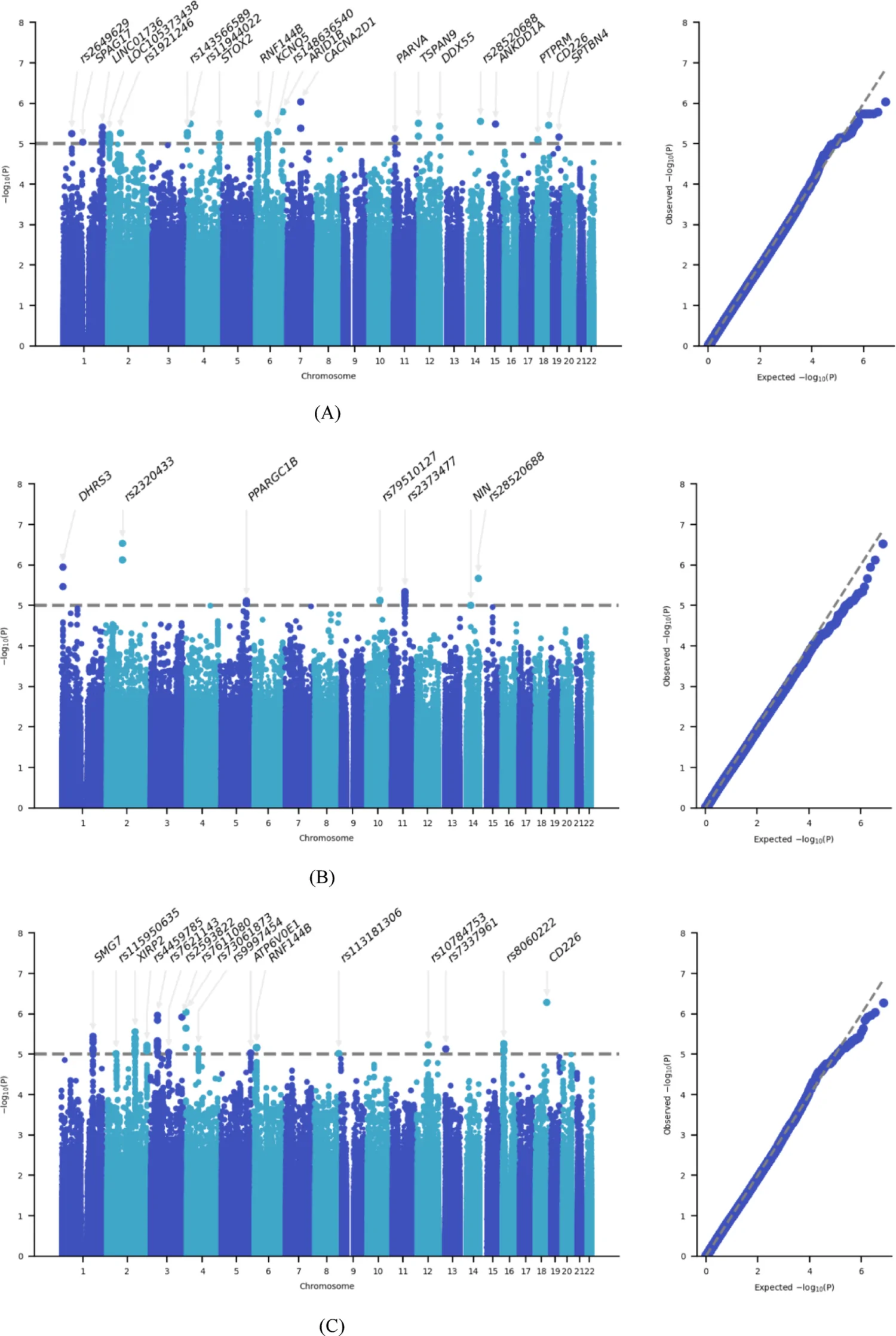
Manhattan plots showing genome-wide association results for children of European-inferred ancestry born before vs. after folic acid fortification and their respective quantile-quantile (QQ) plot and genomic control inflation factor (λ_GC_). **A** GWAS Model 1 includes all individuals of European-inferred ancestry, including CTHD status as a covariate. **B** GWAS Model 2 includes individuals of European-inferred ancestry with CTHD. **C** GWAS Model 3 includes individuals of European-inferred ancestry with other heart defects. The horizontal line denotes the threshold for suggestive significance at *p* = 1 × 10^− 5^. Variants above this line show suggestive association but do not meet the conventional genome-wide significance threshold

No variant reached conventional genome-wide significance (*p* < 5 × 10^− 8^) in any model; all results below are reported at a suggestive threshold. The summary results from the three GWAS models are shown as Manhattan plots, along with the GWAS quantile-quantile plot, in Fig. 2A-C. Overall, genomic inflation was minimal with the first 10 principal components included as covariates (λ_GC_ = 1.009), and LDSC indicated inflation was attributable to polygenicity rather than confounding (intercept = 1.006). Comparable genomic control was observed across all models. Among all study participants (Model 1), 94 variants showed suggestive evidence of association (*p* < 10^− 5^) when comparing all participants born before and after folic acid fortification (Supplemental Table 3). Most variants identified in Model 1 were more frequent prior to folic acid fortification (OR < 1), suggesting a shift whereby these genetic variants were present among individuals with congenital heart defects less frequently following fortification implementation. Variants with suggestive association in Model 1 included multiple variants near *LINC01736* (rs67537171, *p* = 6.47 × 10^− 6^, OR 0.49). In Model 2 (individuals with CTHD), 26 variants showed suggestive evidence of association in individuals born before and after folic acid fortification. Variants located in *DHRS3* (rs7551703 and rs6541043) occurred at higher frequencies among post-fortification births (*p* = 3.43 × 10^− 6^ and 1.13 × 10^− 6^, respectively), suggesting at least two-fold higher odds of the allele being present after fortification (OR 2.10 and 2.21, respectively). Similarly, variants localized to *MIR378A*, an intronic miRNA within the *PPARGC1B* locus (rs251462, rs32589, rs32585, rs32584) were associated with at least a four-fold increase in the odds of occurrence in the post-fortification group (p-values 7.75–8.84 × 10^− 06^, OR 4.47–4.51). In Model 3 (individuals with other heart defects), 59 variants showed suggestive evidence of association in individuals born before or after folic acid fortification. Among these, eight variants within *MIR548A1HG* and one variant within *NKX1-1* showed nominal associations in Models 1 and 3, and all were more frequent among pre-fortification births. The overlap across models suggests that these variants are not restricted to CTHD but may reflect genetic susceptibility shared across broader cardiac phenotypes or driven by non-conotruncal heart defects. Of note, no variants classically associated with folate transport or one-carbon metabolism were detected in any of the models.

**Fig. 3:**
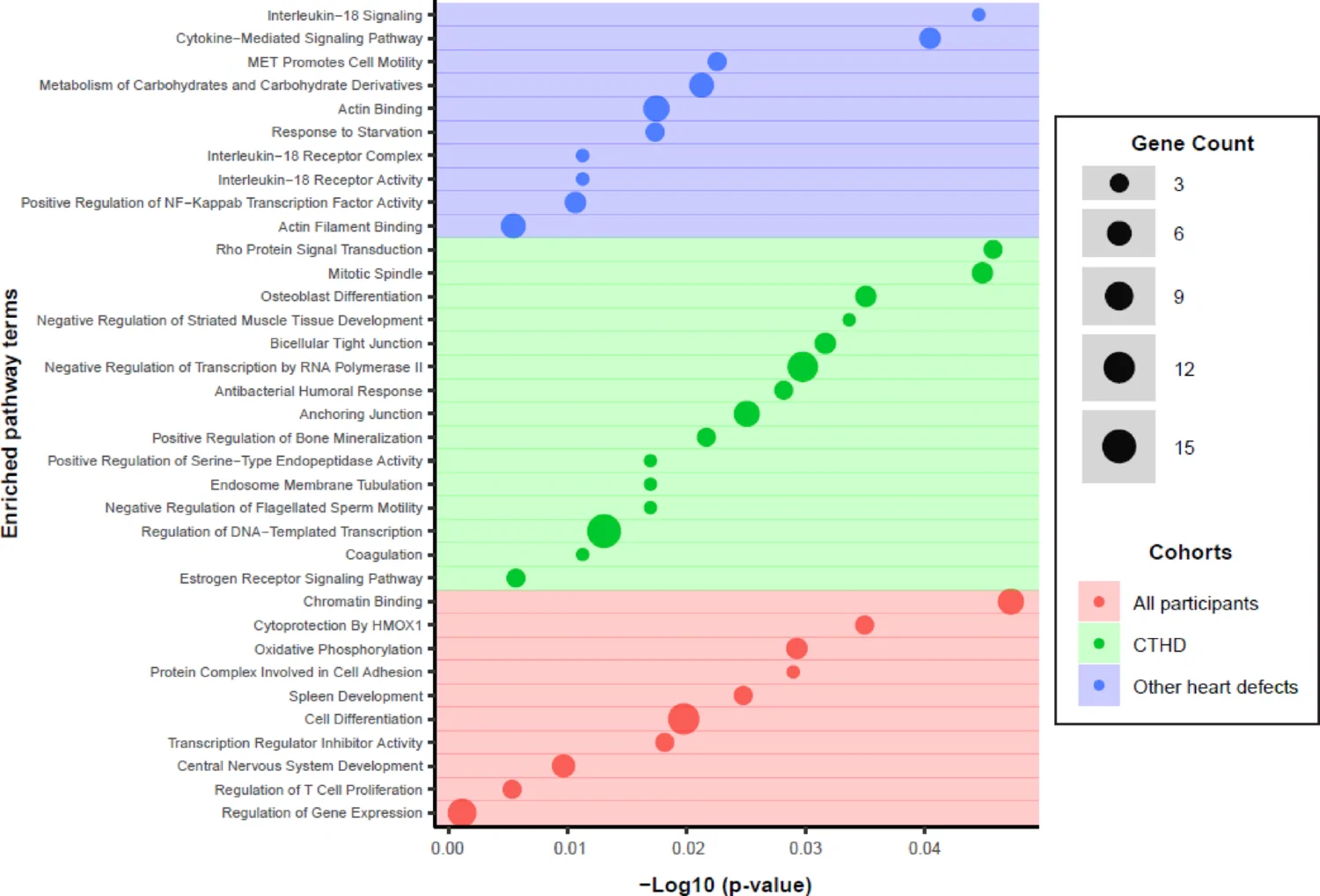
DAVID functional enrichment dot plot across the three GWAS cohorts. Each model shows a distinct set of enriched terms. Dot size reflects the number of genes contributing to each term, and the x-axis represents the -log10 of the geometric mean of the nominal p-values

Functional enrichment was assessed with DAVID across the same three analytic groups and results are summarized by dot plots in Fig. 3. Several functional annotation terms reached nominal significance (unadjusted *p* < 0.05) for each group. Each group exhibited a distinct set of enriched terms (Fig. 3), further suggesting that phenotype-specific shifts in cardiogenetic pathways may have occurred following fortification. Top enriched functional annotation terms in the individuals with CTHD (Model 2) included Rho protein signaling, cytoskeletal dynamics, cell-cell junctions, transcriptional regulation, and intracellular trafficking. These pathway terms are consistent with core developmental processes relevant to heart formation. In individuals with other heart defects (Model 3), top enriched functional annotation terms included chromatin binding, regulation of gene expression, and oxidative phosphorylation, relevant for broader regulatory and metabolic processes. However, because these terms did not remain statistically significant after FDR adjustment, these results should be interpreted as hypothesis-generating rather than confirmatory. A list of all functional annotation terms and p-values for each group are provided in Supplemental Table 4.

## Discussion

Our genome-wide association analysis identified loci in dehydrogenase/reductase 3 (*DHRS3*) and peroxisome proliferator-activated receptor gamma coactivator 1-beta (*PPARGC1B*) among individuals with CTHD that showed differential association with the period of implementation of folic acid fortification in the United States. Among the full analytic group (Model 1) and among individuals with other congenital heart defects (Model 3), a greater number of variants were associated with pre-fortification births compared to among those with CTHD (Model 2), suggesting that observed differences were not CTHD-specific or even lesion-specific.

The broader distribution of suggestively associated variants among all participants (Model 1) and individuals with other congenital heart defects (Model 3) may reflect a change in the genotypic composition of ascertained cases and nutritional shifts following folic acid fortification. Folic acid fortification potentially altered one-carbon metabolism across the entire population during the post-fortification era, influencing methylation and possibly unmasking subtle allele frequency differences. Such widespread changes could generate multiple modest association signals when all participants are analysed together, consistent with a global environmental exposure effect. Interestingly, most of the variants identified in all participants and individuals with other congenital heart defects analyses were more frequent in pre-fortification births and may have become rarer after fortification. This directionality suggests population-level response to increased folic acid exposure. This framework underscores the potential impact of folic acid fortification on population phenotype shift.

For instance, the suggestive associations at *MIR548A1HG* and *NKX1-1* across both Model 1 and 3 indicates that these loci are unlikely to be specific to CTHD and instead represent shared genetic risk factors for broader cardiac phenotypes. Although *NKX1-1* has not been established as a canonical gene in cardiac development, its identification in our pre-fortification GWAS models is biologically intriguing. *NKX1-1* is an NK-family homeobox transcription factor, a class of genes involved in developmental regulation (Simon and Lufkin 2003). Prior cell-free fetal transcriptomic studies have reported its increased expression in the amniotic fluid from fetuses with trisomy 18 and trisomy 21 (Koide et al. 2011), genetic aneuploidies characterized by congenital heart defects. Together, these observations suggest that *NKX1-1* may represent a folate-sensitive regulatory locus but should be interpreted as hypothesis-generating.

In contrast, the more limited number of associated variants among affected individuals suggests that CTHD risk is shaped by a narrower set of loci that interact directly with nutrient-sensitive developmental pathways, although the reason that fewer variants reached the suggestive association threshold within participants with CTHD (Model 2), may also relate more to the CTHD group being smaller and therefore having more limited power. Regardless, the CTHD analysis indicated that carriers of *DHRS3* and *PPARGC1B* alleles occurred at higher frequencies among individuals born after folate fortification, and these genes share biological pathways essential for cardiac morphogenesis and metabolic adaptation, which points toward biologically plausible mechanisms. These findings may support a model in which fortification broadly modified the genomic landscape of folate-responsive loci, while persistent associations in *DHRS3* and *PPARGC1B* represent targeted, biologically focused mechanisms. *DHRS3* is an evolutionarily conserved enzyme that plays a critical regulatory role in embryonic retinoid homeostasis, catalyzing the reduction of all-trans-retinaldehyde to all-trans-retinol, thereby maintaining appropriate intracellular retinoic acid levels during development (Belyaeva et al. 2015). Excessive and deficient amounts of retinoic acid, the active metabolite of vitamin A, and retinoid therapies are well-known teratogens associated with increased risk of spontaneous abortions and birth defects, including congenital heart defects (Enneth et al. 1995). Retinoic acid induces birth defects in animals, namely myelomeningocele, anencephaly, and CTHD (Cohlan 1954; Yasui et al. 1995; Danzer et al. 2005; Cipollone et al. 2006). In the genetic model, ablation of the orthologous gene in multiple species increases retinoic acid levels, with *Dhrs3*^*−/−*^ embryos presenting with defects in cardiac outflow tract formation and atrial and ventricular septation, along with defects in skeletal, and craniofacial development (Danzer et al. 2005; Billings et al. 2013). In humans, inherited variants in *DHRS3* have been reported in families presenting with phenotypic features of craniosynostosis, facial dysmorphism, congenital heart defects (observed in 4 out of 5 affected individuals), and scoliosis (Hashimoto et al. 2025). Importantly, maternal folate deficiency is independently linked to an increased risk of neural tube defects and congenital heart defects (Czeizel 1993; Czeizel et al. 2013), and experimental models demonstrate that folic acid supplementation rescues neural tube defects, cleft palate, and congenital heart defects induced by retinoic acid or its antagonist (Reynolds et al. 2003; Cipollone et al. 2009; Wu et al. 2020). More recent work indicates that vitamin A and folic acid act in concert during neural tube closure, offering one explanation for the subset of neural tube defects that remain refractory to folic acid fortification (Edri et al. 2026). These studies have been instrumental in establishing the role of prenatal vitamin supplementation in birth defect prevention.

PPARGC1β encodes PGC-1β, a transcriptional coactivator that regulates cardiac development and mitochondrial gene expression (Rowe et al. 2010; Murphy et al. 2021), particularly in models of heart failure (Sihag et al. 2009). PGC-1α has been studied more extensively, but PGC-1α and PGC-1β share functional overlap as coactivators of nuclear receptors, including the PPARs, in metabolic regulation and mitochondrial biogenesis. Notably, the four associated variants localize to *MIR378A*, an intronic miRNA within the *PPARGC1B* locus. miR-378a is co-transcribed with *PPARGC1B* and has been implicated in the regulation of cardiac (Krist et al. 2015) and hepatic energy metabolism (Xu et al. 2023), providing an alternative candidate mechanism at this locus. Because the associated variants are in linkage disequilibrium across the region, the present data cannot resolve whether *PPARGC11B*,* MIR378A*, or another regulatory element accounts for the signal. The role of PGC-1β is best established during the postnatal period when it activates the metabolic and functional maturation of the heart. In loss-of-function studies, *Pgc-1α*^−/−^*Pgc-1β*^−/−^ mice display structurally normal but smaller hearts and exhibit conduction abnormalities such as bradycardia and heart block (Lai et al. 2008). The developmental timing of PGC-1β activity during late fetal and perinatal cardiac development argues against a direct interaction with maternal folate status during early embryogenesis. We therefore consider a non-mechanistic explanation more likely for this locus. Over the study period, advances in prenatal diagnosis, intensive care, and surgical management substantially improved survival among children with CTHD (Oster et al. 2013), and PCGC enrollment requires survival to ascertainment. Variants that predispose to impaired metabolic activity or heart failure could therefore have become more frequent among surviving, enrolled children in the post-fortification era. This survival-and-ascertainment explanation is not testable in the present dataset. The contrast between these two loci is informative: *DHRS3* aligns with the cardiac developmental window and known folate-retinoid interaction, whereas *PPARGC1B* is better explained by temporal changes in survival and ascertainment. We therefore regard *DHRS3* as the stronger candidate for replication.(Oster et al. 2013).

Strengths of our association study on the impact of folate supplementation on CTHD include the integration of the United States folic acid fortification policy into the study design. We accounted for the phased implementation and adoption of fortification during pregnancy by incorporating a two-year washout period, thereby improving classification of the two exposure groups. Additionally, we utilized existing high-quality genomic data from a well-phenotyped cohort available through the PCGC, enabling analysis of a relatively large population with CTHD to maximize statistical power. A key design consideration was the use of participants with other congenital heart defects from the same multicenter study network as the reference group, rather than unaffected controls. This approach was intended to minimize bias related to healthcare access and ascertainment. Finally, rigorous QC measures, including restriction to individuals of European-inferred ancestry, minimized population stratification and reduced confounding due to genetic heterogeneity.

Limitations of our study include potential phenotypic heterogeneity among all of our analytic groups, as specific lesions may differ in their underlying genetic or environmental etiologies, which is a common limitation in GWAS of congenital heart defects. In this exploratory study, we also reported suggestive associations that need confirmation in larger independent cohorts as they become available. The use of a less stringent significance threshold increases the possibility of false-positive associations, and thus, the results should be interpreted cautiously and validated in independent cohorts. The absence of detailed, individual-level data on periconceptional maternal diet and supplement use prevented assessment of individual-level folate exposure, and therefore, the effects of fortification may vary according to the baseline nutritional status of the individual. In fact, observed differences between pre- and post-fortification cohorts may partially reflect secular trends unrelated to fortification, such as improved prenatal detection and survival of complex lesions or population changes in maternal characteristics (e.g., obesity) and behaviors (e.g., smoking) over time rather than directly relating to fortification policy. Although replication in independent populations is warranted, the PCGC remains one of the largest of United States congenital heart defects cohorts with genomic data. Additionally, restricting analyses to individuals of European-inferred ancestry reduces confounding from population stratification but limits the generalizability of findings to other ancestral groups. Our study also focused on the offspring genetic effect rather than the maternal genetic effect, and future work is needed to better understand potential changes in associated maternal variants following fortification. Despite these limitations, the study offers valuable insights into potential shifts in pathways and genetic factors among individuals with CTHD and other congenital heart defects following the era of fortification.

## Conclusion

In summary, this study integrates genomic and temporal data to provide insights into how folic acid fortification policy may have influenced genetic risk for CTHD. Our study captures this historical policy milestone as a natural experiment to examine folate-genotype interactions. Importantly, these findings extend beyond a historical policy context as mandatory folic acid fortification remains absent in many countries, meaning the gene-nutrient interactions identified here may reflect nutritional conditions still experienced by the global majority. By leveraging a large, well-phenotyped cohort, we identified potential gene-nutrient interactions that may modulate cardiac developmental pathways. These findings highlight the complex interplay between maternal folate status and embryonic retinoid levels in shaping cardiovascular development. Continued investigation into gene-nutrient interactions may inform targeted prevention strategies and refine our understanding of how population-level nutritional policies may alter the penetrance of genetic susceptibility and, in turn, the genotypic composition of affected populations.

## Data Availability

The datasets generated during and/or analysed during the current study are available in the Genotypes and Phenotypes (dbGaP) repository, https://dbgap.ncbi.nlm.nih.gov/beta/study/phs000571.v7.p3/#study.

## References

[CR1] Agopian AJ, Mitchell LE, Glessner J, Bhalla AD, Sewda A, Hakonarson H, Goldmuntz E (2014) Genome-wide association study of maternal and inherited loci for conotruncal heart defects. PLoS One. 10.1371/journal.pone.0096057 PMC4011736 24800985

[CR2] Agopian AJ, Goldmuntz E, Hakonarson H, Sewda A, Taylor D, Mitchell LE (2017) Genome-wide association studies and meta-analyses for congenital heart defects. Circ Cardiovasc Genet. 10.1161/CIRCGENETICS.116.001449 PMC5539991 28468790

[CR3] Bedard T, Lowry RB, Sibbald B, Harder JR, Trevenen C, Horobec V, Dyck JD (2013) Folic acid fortification and the birth prevalence of congenital heart defect cases in Alberta, Canada. Birth Defects Res A Clin Mol Teratol 97(8):564–570. 10.1002/bdra.23162 23913528

[CR4] Belyaeva OV, Chang C, Berlett MC, Kedishvili NY (2015) Evolutionary origins of retinoid active short-chain dehydrogenases/reductases of SDR16C family. Chem Biol Interact 234:135–143. 10.1016/j.cbi.2014.10.026 PMC4414673 25451586

[CR5] Billings SE, Pierzchalski K, Tjaden NEB, Pang XY, Trainor PA, Kane MA, Moise AR (2013) The retinaldehyde reductase DHRS3 is essential for preventing the formation of excess retinoic acid during embryonic development. FASEB J 27(12):4877–4889. 10.1096/fj.13-227967 PMC3834788 24005908

[CR6] Botto LD, Mum,;, Khoury J, Mulinare J, Erickson J-D (1996) *Periconceptional Multivitamin Use and the Occurrence of Conotruncal Heart Defects: Results From a Population-based, Case-Control Study*, *PEDIATRICS*. Available at: http://publications.aap.org/pediatrics/article-pdf/98/5/911/999930/911.pdf 8909485

[CR7] Botto LD, Lin AE, Riehle-Colarusso T, Malik S, Correa A, National Birth Defects Prevention Study (2007) Seeking causes: classifying and evaluating congenital heart defects in etiologic studies. Birth defects research. Part A, Clinical and molecular teratology 79(10):714–27. 10.1002/bdra.20403 17729292

[CR8] Cipollone D, Amati F, Carsetti R, Placidi S, Biancolella M, D’Amati G, Novelli G, Siracusa G, Marino B (2006) A multiple retinoic acid antagonist induces conotruncal anomalies, including transposition of the great arteries, in mice. Cardiovascular Pathology. 10.1016/j.carpath.2006.04.004 16844550

[CR9] Cipollone D, Carsetti R, Tagliani A, Rosado MM, Borgiani P, Novelli G, D’Amati G, Fumagalli L, Marino B, Businaro R (2009) Folic acid and methionine in the prevention of teratogen-induced congenital defects in mice. Cardiovascular Pathology. 10.1016/j.carpath.2008.02.007 18417366

[CR10] Cohlan SQ (1954) Congenital anomalies in the rat produced by excessive intake of vitamin A during pregnancy. Pediatrics 13(6):556–567 13166454

[CR11] Czeizel AE (1993) Prevention of congenital abnormalities by periconceptional multivitamin supplementation. BMJ 306(6893):1645–1648. 10.1136/bmj.306.6893.1645 PMC1678103 8324432

[CR12] Czeizel AE, Dudás I, Vereczkey A, Bánhidy F (2013) Folate deficiency and folic acid supplementation: The prevention of neural-tube defects and congenital heart defects. Nutri. 10.3390/nu5114760 PMC3847759 24284617

[CR13] Czeizel AE, Vereczkey A, Szabó I (2015) Folic acid in pregnant women associated with reduced prevalence of severe congenital heart defects in their children: a national population-based case-control study. Eur J Obstet Gynecol Reprod Biol 193:34–9. 10.1016/j.ejogrb.2015.06.024 26225846

[CR14] Danzer E, Schwarz U, Wehrli S, Radu A, Adzick NS, Flake AW (2005) Retinoic acid induced myelomeningocele in fetal rats: Characterization by histopathological analysis and magnetic resonance imaging. *Experimental Neurology*. 10.1016/j.expneurol.2005.03.011 15893307

[CR15] Dietrich M, Brown CJP, Block G (2005) The effect of folate fortification of cereal-grain products on blood folate status, dietary folate intake, and dietary folate sources among adult non-supplement users in the United States. J American College Nutri. 10.1080/07315724.2005.10719474 16093404

[CR16] Enneth K, Othman JR, Oore YLM, Inger ARS, Guyen Y-SADTN, Alvatore S, Annino M (1995) Teratogenicity of high vitamin A intake. N Engl J Med 333:1369–1373 10.1056/NEJM199511233332101 7477116

[CR17] Edri T, Abbou-Levy T, Cohen D, Inacio JM, Shabtai Y, Pillemer G, Belo JA, Fainsod A (2026) Folic acid prevention of neural tube defects requires retinoic acid produced by ALDH1L1, *Proc Natl Acad Sci U S A.*, 123(31). Available at: 10.1073/pnas PMC13438623 42531027

[CR18] Feng Y, Wang S, Chen R, Tong X, Wu Z, Mo X (2015) Maternal folic acid supplementation and the risk of congenital heart defects in offspring: A meta-analysis of epidemiological observational studies. *Scientific Reports*. 10.1038/srep08506 PMC4330542 25687545

[CR19] Goldmuntz E, Woyciechowski S, Renstrom D, Lupo PJ, Mitchell LE (2008) Variants of folate metabolism genes and the risk of conotruncal cardiac defects. Circ Cardiovasc Genet 1(2):126–32. 10.1161/CIRCGENETICS.108.796342 PMC3035562 20031554

[CR20] Hanchard NA, Swaminathan S, Bucasas K, Furthner D, Fernbach S, Azamian MS, Wang X, Lewin M, Towbin JA, D’Alessandro LCA, Morris SA, Dreyer W, Denfield S, Ayres NA, Franklin WJ, Justino H, Lantin-Hermoso MR, Ocampo EC, Santos AB, Parekh D, Moodie D, Jeewa A, Lawrence E, Allen HD, Penny DJ, Fraser CD, Lupski JR, Popoola M, Wadhwa L, Brook JD, Bu’Lock FA, Bhattacharya S, Lalani SR, Zender GA, Fitzgerald-Butt SM, Bowman J, Corsmeier D, White P, Lecerf K, Zapata G, Hernandez P, Goodship JA, Garg V, Keavney BD, Leal SM, Cordell HJ, Belmont JW, McBride KL (2016) A genome-wide association study of congenital cardiovascular left-sided lesions shows association with a locus on chromosome 20. Human molecular genetics. 10.1093/hmg/ddw071 PMC5081047 26965164

[CR21] Hashimoto AS, Yu J, Williams C, Gaudenz K, Varshosaz P, Zhao R, Pilli N, Liu T, Russell J, Tooze RS, Twigg SRF, Banka S, Sweeney E, McGowan SJ, Knight SJL, Taylor JC, Froukh TJ, Palafoll MIV, Martínez-Gil N, Costa-Roger M, Villarreal-Molina MT, Hernandez L, Jamra EA, Gattermann R, Koch-Hogrebe F, Wieczorek M, Trainor D, Moise PA, Wilkie AR, Kane MA (2025) Identification and characterization of short-chain dehydrogenase/reductase 3 (DHRS3) deficiency, a retinoic acid embryopathy of humans. Genetics in Medicine Open. 10.1016/j.gimo.2025.103427 PMC12167057 40519748

[CR22] Hazelett DJ, Rhie SK, Gaddis M, Yan C, Lakeland DL, Coetzee SG, Henderson BE, Noushmehr H, Cozen W, Kote-Jarai Z, Eeles RA, Easton DF, Haiman CA, Lu W, Farnham PJ, Coetzee GA (2014) Comprehensive functional annotation of 77 prostate cancer risk loci. PLoS Genet 10(1):e1004102. 10.1371/journal.pgen.1004102 PMC3907334 24497837

[CR23] Hobbs CA, James SJ, Jernigan S, Melnyk S, Lu Y, Malik S, Cleves MA (2006) Congenital heart defects, maternal homocysteine, smoking, and the 677 C > T polymorphism in the methylenetetrahydrofolate reductase gene: evaluating gene-environment interactions. American journal of obstetrics and gynecology. 10.1016/j.ajog.2005.06.016 16389035

[CR24] Hobbs CA, MacLeod SL, Jill James S, Cleves MA (2011) Congenital heart defects and maternal genetic, metabolic, and lifestyle factors. Birth defects research. 10.1002/bdra.20784 21384532

[CR25] Hu Z, Shi Y, Mo X, Xu J, Zhao B, Lin Y, Yang S, Xu Z, Dai J, Pan S, Da M, Wang X, Qian B, Wen Y, Wen J, Xing J, Guo X, Xia Y, Ma H, Jin G, Yu S, Liu J, Zhou Z, Wang X, Chen Y, Sha J, Shen H (2013) A genome-wide association study identifies two risk loci for congenital heart malformations in Han Chinese populations. Nat Genet 45(7):818–21. 10.1038/ng.2636 23708190

[CR26] Huang DW, Sherman BT, Lempicki RA (2009) Systematic and integrative analysis of large gene lists using DAVID bioinformatics resources. Nat Protoc 4(1):44–57. 10.1038/nprot.2008.211 19131956

[CR27] Koide K, Slonim DK, Johnson KL, Tantravahi U, Cowan JM, Bianchi DW (2011) Transcriptomic analysis of cell-free fetal RNA suggests a specific molecular phenotype in trisomy 18. Hum Genet 129(3):295–305. 10.1007/s00439-010-0923-3 PMC3206603 21152935

[CR28] Krist B, Florczyk U, Pietraszek-Gremplewicz K, Józkowicz A, Dulak J (2015) The role of miR-378a in metabolism, angiogenesis, and muscle biology. International Journal of Endocrinology. 10.1155/2015/281756 PMC4709675 26839547

[CR29] Lahm H, Jia M, Dreßen M, Wirth F, Puluca N, Gilsbach R, Keavney BD, Cleuziou J, Beck N, Bondareva O, Dzilic E, Burri M, König KC, Ziegelmüller JA, Abou-Ajram C, Neb I, Zhang Z, Doppler SA, Mastantuono E, Lichtner P, Eckstein G, Hörer J, Ewert P, Priest JR, Hein L, Lange R, Meitinger T, Cordell HJ, Müller-Myhsok B, Krane M (2021) Congenital heart disease risk loci identified by genome-wide association study in European patients. J Clin Invest. 10.1172/JCI141837 PMC7810487 33201861

[CR30] Lai L, Leone TC, Zechner C, Schaeffer PJ, Kelly SM, Flanagan DP, Medeiros DM, Kovacs A, Kelly DP (2008) Transcriptional coactivators PGC-lα and PGC-lβ control overlapping programs required for perinatal maturation of the heart. Genes Dev 22(14):1948–1961. 10.1101/gad.1661708 PMC2492740 18628400

[CR31] Leirgul E, Fomina T, Brodwall K, Greve G, Holmstrøm H, Vollset SE, Tell GS, Øyen N (2014) Birth prevalence of congenital heart defects in Norway 1994–2009 - A nationwide study. American Heart Journal. 10.1016/j.ahj.2014.07.030 25458661

[CR32] Li W, Shih A, Freudenberg-Hua Y, Fury W, Yang Y (2021) Beyond standard pipeline and p < 0.05 in pathway enrichment analyses. Comput Biol Chem 92:107455. 10.1016/j.compbiolchem.2021.107455 PMC9179938 33774420

[CR33] Van Der Linde D, Konings EEM, Slager MA, Witsenburg M, Helbing WA, Takkenberg JJM, Roos-Hesselink JW (2011) Birth prevalence of congenital heart disease worldwide: a systematic review and meta-analysis. J American College Cardiology. 10.1016/j.jacc.2011.08.025 22078432

[CR34] Lupo PJ, Goldmuntz E, Mitchell LE (2010) Gene-gene interactions in the folate metabolic pathway and the risk of conotruncal heart defects. J Biomed Biotechnol 2010:630940. 10.1155/2010/630940 PMC2810479 20111745

[CR35] Mamasoula C, Prentice RR, Pierscionek T, Pangilinan F, Mills JL, Druschel C, Pass K, Russell MW, Hall D, Töpf A, Brown DL, Zelenika D, Bentham J, Cosgrove C, Bhattacharya S, Riveron JG, Setchfield K, Brook JD, Bu’Lock FA, Thornborough C, Rahman TJ, Doza JP, Tan HL, O’Sullivan J, Stuart AG, Blue G, Winlaw D, Postma AV, Mulder BJM, Zwinderman AH, van Engelen K, Moorman AFM, Rauch A, Gewillig M, Breckpot J, Devriendt K, Lathrop GM, Farrall M, Goodship JA, Cordell HJ, Brody LC, Keavney BD (2013) Association between C677T polymorphism of methylene tetrahydrofolate reductase and congenital heart disease: meta-analysis of 7697 cases and 13 125 controls. Circulation: Cardiovascular Genetics. 10.1161/CIRCGENETICS.113.000191 PMC3855044 23876493

[CR36] Mitchell LE, Agopian AJ, Bhalla A, Glessner JT, Kim CE, Swartz MD, Hakonarson H, Goldmuntz E (2015) Genome-wide association study of maternal and inherited effects on left-sided cardiac malformations. Human Molecular Genetics. 10.1093/hmg/ddu420 PMC4326325 25138779

[CR37] Murphy SA, Miyamoto M, Kervadec A, Kannan S, Tampakakis E, Kambhampati S, Lin BL, Paek S, Andersen P, Lee DI, Zhu R, An SS, Kass DA, Uosaki H, Colas AR, Kwon C (2021) PGC1/PPAR drive cardiomyocyte maturation at single cell level via YAP1 and SF3B2. Nat Commun. 10.1038/s41467-021-21957-z PMC7955035 33712605

[CR38] National Academies of Sciences, E. and M. (2023) Using population descriptors in genetics and genomics research: A new framework for an evolving field. The National Academies Press, Washington, DC 36989389

[CR39] Oster ME, Lee KA, Honein MA, Riehle-Colarusso T, Shin M, Correa A (2013) Temporal trends in survival among infants with critical congenital heart defects. Pediatrics. 10.1542/peds.2012-3435 PMC4471949 23610203

[CR40] Pediatric Cardiac Genomics Consortium, Gelb B, Brueckner M, Chung W, Goldmuntz E, Kaltman J, Kaski JP, Kim R, Kline J, Mercer-Rosa L, Porter G, Roberts A, Rosenberg E, Seiden H, Seidman C, Sleeper L, Tennstedt S, Kaltman J, Schramm C, Burns K, Pearson G, Rosenberg E (2013) he congenital heart disease genetic network study: rationale, design, and early results. Circulation research. 10.1161/CIRCRESAHA.111.300297 PMC3679175 23410879

[CR41] Qu Y, Lin S, Zhuang J, Bloom MS, Smith M, Nie Z, Mai J, Ou Y, Wu Y, Gao X, Tan H, Liu X (2020) First-trimester maternal folic acid supplementation reduced risks of severe and most congenital heart diseases in offspring: a large case-control study. J Am Heart Assoc. 10.1161/JAHA.119.015652 PMC7670504 32613868

[CR42] Raggi F, Cangelosi D, Becherini P, Blengio F, Morini M, Acquaviva M, Belli ML, Panizzon G, Cervo G, Varesio L, Eva A, Bosco MC (2020) Transcriptome analysis defines myocardium gene signatures in children with ToF and ASD and reveals disease-specific molecular reprogramming in response to surgery with cardiopulmonary bypass. J Transl Med 18(1):21. 10.1186/s12967-020-02210-5 PMC6954611 31924244

[CR43] Reynolds PR, Schaalje GB, Seegmiller RE (2003) Effect of arsenite, maternal age, and embryonic sex on spina bifida, exencephaly, and resorption rates in the splotch mouse. Birth Defects Research Part A: Clinical and Molecular Teratology. 10.1002/bdra.10036 12854658

[CR44] Richter F, Morton SU, Kim SW, Kitaygorodsky A, Wasson LK, Chen KM, Zhou J, Qi H, Patel N, DePalma SR, Parfenov M, Homsy J, Gorham JM, Manheimer KB, Velinder M, Farrell A, Marth G, Schadt EE, Kaltman JR, Newburger JW, Giardini A, Goldmuntz E, Brueckner M, Kim R, Porter GA, Bernstein D, Chung WK, Srivastava D, Tristani-Firouzi M, Troyanskaya OG, Dickel DE, Shen Y, Seidman JG, Seidman CE, Gelb BD (2020) Genomic analyses implicate noncoding de novo variants in congenital heart disease. Nat Genet 52(8):769–777. 10.1038/s41588-020-0652-z PMC7415662 32601476

[CR45] Rosenquist TH (2013) Folate, homocysteine and the cardiac neural crest. Dev Dyn 242(3):201–18. 10.1002/dvdy.23922 23335187

[CR46] Rowe GC, Jiang A, Arany Z (2010) PGC-1 coactivators in cardiac development and disease. Circ Res 107(7):825–838. 10.1161/CIRCRESAHA.110.223818 PMC2955978 20884884

[CR47] Shaw GM, Zhu H, Lammer EJ, Yang W, Finnell RH (2003) Genetic variation of infant reduced folate carrier (A80G) and risk of orofacial and conotruncal heart defects. American journal of epidemiology. 10.1093/aje/kwg189 14561664

[CR48] Sherman BT, Hao M, Qiu J, Jiao X, Baseler MW, Lane HC, Imamichi T, Chang W (2022) DAVID: a web server for functional enrichment analysis and functional annotation of gene lists (2021 update). Nucleic Acids Res 50(W1):W216–W221. 10.1093/nar/gkac194 PMC9252805 35325185

[CR49] Sihag S, Cresci S, Li AY, Sucharov CC, Lehman JJ (2009) PGC-1α and ERRα target gene downregulation is a signature of the failing human heart. J Mol Cell Cardiol 46(2):201–212. 10.1016/j.yjmcc.2008.10.025 PMC2681265 19061896

[CR50] Simon R, Lufkin T (2003) Postnatal lethality in mice lacking the Sax2 homeobox gene homologous to *Drosophila* S59/slouch: evidence for positive and negative autoregulation. Mol Cell Biol 23(24):9046–9060. 10.1128/mcb.23.24.9046-9060.2003 PMC309705 14645517

[CR51] Tang LS, Wlodarczyk BJ, Santillano DR, Miranda RC, Finnell RH (2004) Developmental consequences of abnormal folate transport during murine heart morphogenesis. Birth Defects Research Part A: Clinical and Molecular Teratology. 10.1002/bdra.20043 15259034

[CR52] Taparia S, Gelineau-van Waes J, Rosenquist TH, Finnell RH (2007) Importance of folate-homocysteine homeostasis during early embryonic development. Clinical chemistry and laboratory medicine. 10.1515/CCLM.2007.345 18067451

[CR53] Theis JL, Shatila SDH, Fogarty ZC, Bamlet WR, Olson TM (2023) Genome-wide association and inheritance-based analyses implicate unconventional myosin genes in hypoplastic left heart syndrome. Circ Genom Precis Med 16(1):E003761. 10.1161/CIRCGEN.122.003761 36580305

[CR54] Wang B, Liu M, Yan W, Mao J, Jiang D, Li H, Chen Y (2013) Association of SNPs in genes involved in folate metabolism with the risk of congenital heart disease. J Matern Fetal Neonatal Med 26(18):1768–77. 10.3109/14767058.2013.799648 23701284

[CR55] Wu F, Gu C, Bi Y, Guo Z, Wang Y (2020) Folic acid rescues all-trans retinoic acid-induced anorectal malformations in rats. Birth Defects Research. 10.1002/bdr2.1810 33459508

[CR56] Xu J, Cui L, Wang J, Zheng S, Zhang H, Ke S, Cao X, Shi Y, Li J, Zen K, Vidal-Puig A, Zhang CY, Li L, Jiang X (2023) Cold-activated brown fat-derived extracellular vesicle-miR-378a-3p stimulates hepatic gluconeogenesis in male mice. Nat Commun. 10.1038/s41467-023-41160-6 PMC10482845 37673898

[CR57] Yasui H, Nakazawa M, Morishima M, Miyagawa-Tomita S, Momma K (1995) Morphological observations on the pathogenetic process of transposition of the great arteries induced by retinoic acid in mice. Circulation. 10.1161/01.cir.91.9.2478 7729035

[CR58] Zhu H, Yang W, Shaw N, Perloff S, Carmichael SL, Finnell RH, Shaw GM, Lammer EJ (2012) Thymidylate synthase polymorphisms and risk of conotruncal heart defects. American J Med Genetics Part A. 10.1002/ajmg.a.35310 PMC3429723 22887475

